# Learning to push and learning to move: the adaptive control of contact forces

**DOI:** 10.3389/fncom.2015.00118

**Published:** 2015-11-06

**Authors:** Maura Casadio, Assaf Pressman, Ferdinando A. Mussa-Ivaldi

**Affiliations:** ^1^Informatics, Bioengineering, Robotics and Systems Engineering, University of GenoaGenoa, Italy; ^2^Department of Physiology, Northwestern UniversityChicago, IL, USA; ^3^Robotics, Brain and Cognitive Sciences, Istituto Italiano di TecnologiaGenoa, Italy; ^4^Department of Biomedical Engineering, Ben Gurion University of the NegevBe'er Sheva, Israel; ^5^Sensory Motor Performance Program, Rehabilitation Institute of ChicagoChicago, IL, USA

**Keywords:** force control, motor learning, modularity, impedance, kinematics, dynamics of manual skill

## Abstract

To be successful at manipulating objects one needs to apply simultaneously well controlled movements and contact forces. We present a computational theory of how the brain may successfully generate a vast spectrum of interactive behaviors by combining two independent processes. One process is competent to control movements in free space and the other is competent to control contact forces against rigid constraints. Free space and rigid constraints are singularities at the boundaries of a continuum of mechanical impedance. Within this continuum, forces and motions occur in “compatible pairs” connected by the equations of Newtonian dynamics. The force applied to an object determines its motion. Conversely, inverse dynamics determine a unique force trajectory from a movement trajectory. In this perspective, we describe motor learning as a process leading to the discovery of compatible force/motion pairs. The learned compatible pairs constitute a local representation of the environment's mechanics. Experiments on force field adaptation have already provided us with evidence that the brain is able to predict and compensate the forces encountered when one is attempting to generate a motion. Here, we tested the theory in the dual case, i.e., when one attempts at applying a desired contact force against a simulated rigid surface. If the surface becomes unexpectedly compliant, the contact point moves as a function of the applied force and this causes the applied force to deviate from its desired value. We found that, through repeated attempts at generating the desired contact force, subjects discovered the unique compatible hand motion. When, after learning, the rigid contact was unexpectedly restored, subjects displayed after effects of learning, consistent with the concurrent operation of a motion control system and a force control system. Together, theory and experiment support a new and broader view of modularity in the coordinated control of forces and motions.

## Introduction

When writing and drawing we must push the pencil hard enough against the paper to produce a steady trace, but lightly enough to avoid breaking the brittle graphite tip. Many other daily activities require coordinated combinations of motions and contact forces.

Studies of multi-unit activities in posterior-parietal cortex have revealed the existence of neural structures that appear to be selectively involved in the geometric planning of movement (Snyder et al., 1997; Buneo and Andersen, 2006; Torres and Andersen, 2006), but not in the control of the forces that underlie movement execution. Kalaska and coworkers (Hamel-Paquet et al., 2006) have found neurons in the posterior parietal cortex area 5 that show tuning for the direction of movement, but not for the direction of hand-applied isometric force. The same authors (Sergio and Kalaska, 1998; Sergio et al., 2005) also described systematic differences in the activities of M1 neurons during movement tasks and isometric tasks.

While motions and forces are likely to have separate neural representations, they need to be controlled concurrently in tasks involving mechanical interactions with the external environment^1^. A well-known example of concurrent force and motion control is offered by the adaptation of arm movements to predictable force perturbations (Flash and Gurevich, 1992; Lackner and Dizio, 1994; Shadmehr and Mussa-Ivaldi, 1994; Gandolfo et al., 1996; Conditt et al., 1997; Thoroughman and Shadmehr, 2000; Scheidt et al., 2001). The brain, as an adaptive control system, learns to generate forces that cancel the external field. Is there a similar adaptive mechanism when the task is to generate a force against a perturbing motion? In this case, a symmetric—or “dual”—mechanism would call for the generation of compensatory motions to restore the desired force profile. In analogy with movement adaptation to force fields, we expect that the corresponding adaptive process for the coordinated production of contact force in the presence of predictable motions would result in a local representation of the environment mechanics. This representation would be based on the identification of causal connections between movement and force trajectories within the respective experienced domains.

In principle, a desired behavior can be enforced by pure feedback mechanisms, without forming a representation of the perturbing environment. When the environment is not predictable, the maintenance of a desired motion or a desired contact force by feedback mechanisms can be accomplished by shifting the interface impedance toward two opposing limits. In motion control, random forces are counteracted by high position feedback gains, resulting in high contact impedance. In force control, random motions are compensated by increasing force feedback gains, resulting in low impedance. In the biological system, the neural controller can modulate contact impedance by varying the level of muscle coactivation (Mussa-Ivaldi et al., 1985) and by exploiting the kinematic redundancy of the limbs (Hogan, 1985a). Furthermore, impedance is also regulated—at least partially—by active mechanisms based on sensory feedback. However, the possibility to shift impedance toward high or low values is constrained by the passive mechanics of muscles and bones and by the long neural transmission delays. Because of these intrinsic limitations to biological feedback control, the brain may exploit the predictable properties of the environment to modify accordingly the “feedforward” commands. The ability to do so has been demonstrated for the predictive compensation of deterministic force fields during reaching movements of the arm (Shadmehr and Mussa-Ivaldi, 1994) and of the leg (Emken and Reinkensmeyer, 2005). In this study we investigated the possibility that a similar mechanism may facilitate the control of contact forces against deterministic motions. We consider this issue within a theoretical framework that extends the concept of internal models and unifies the approach to the motor learning of forces and motions.

We developed a computational theory that integrates the control of motion and forces by combining two independent functional modules. One is competent to generate arbitrary motions in free space. The other is competent to generate arbitrary forces against rigid surfaces. Together, these modules can produce—by simply adding their outputs—a broad repertoire of adaptive behaviors in several mechanical environments, represented by state-determined models. In this framework, both force and motion variables may be concurrently specified along non-orthogonal directions. This is similar to the parallel control of Chiaverini, Sciavicco, and Siciliano (Chiaverini and Sciavicco, 1993; Siciliano, 1996). A key prediction of our theory is that after subjects have adapted the control of force to the transition from rigid to soft contact, the sudden reversal to a rigid contact would cause a sizable after effect.

## Materials and methods

### A theory of motor adaptation

The laws of Newton insure that when we apply a force to an object, the object moves along a unique trajectory. Conversely, to move an object along a given trajectory we must apply a unique, well-defined force profile. While apparently trivial, these two statements are not always true. When we apply a normal force upon a wall, i.e., an infinitely stiff environment, we have a particular movement trajectory—x(t) = constant—associated with an infinite variety of possible contact force trajectories^2^. In a related—but opposite or “dual”—condition, when we move the hand in free space, i.e., in an infinitely compliant environment, infinite possible trajectories can be paired with a single applied (near) zero- contact-force trajectory at the interface between the hand and the virtually massless air. Free space and rigid constraints are two extreme—or “singular”—cases, between which lies a continuum of regular environments, where force and movement trajectories come only in “mutually compatible” pairs: a force trajectory determines a unique movement, the solution of an ordinary differential equation, and vice-versa time history of position, velocity and acceleration determines a unique force trajectory, the solution of an algebraic identity.

We make the hypothesis that the brain is competent to operate in the two singular conditions: it is capable to produce desired movements of the limbs in free space and to apply forces of various amplitude, direction and temporal profile against rigid contacts. Starting from these two basic skills, learning comes into play when the brain faces new non-singular environments. The concurrent operation of force and motion control leads to the formation of what some authors (Shadmehr and Mussa-Ivaldi, 1994; Kawato and Wolpert, 1998) have called an “internal model” of the environment. Here, we propose a new interpretation of this concept. We consider the internal model as a computational process that explicitly seeks the motion (force) trajectory compatible with the planned force (motion) trajectory within non-singular environments, which establish a coupling between feasible forces and motions.

In this perspective, the experiments on force field adaptation (Shadmehr and Mussa-Ivaldi, 1994; Gandolfo et al., 1996; Conditt et al., 1997; Thoroughman and Shadmehr, 2000) are particular instances, in which the brain recovers a desired movement trajectory by producing a force trajectory that cancels the perturbing field. Starting from these known results, we now propose that the brain also accomplishes the dual outcome of producing a desired force trajectory against a “soft” environment by finding and generating the unique movement that the environment associates to the desired force.

If one is applying a desired force against a rigid contact and the contact suddenly becomes compliant, one observes an unexpected change both in position and contact force. There are two options in this case for maintaining the desired force amplitude and direction: (a) changing the desired position based on the observed motion of the contact point, or (b) changing the desired force, based on the observed force error. Both approaches would converge to the same end result, i.e., to the unique pair of force and motion trajectories, compatible with the impedance of the soft contact. Here, we pursued the first approach based on the consideration that the accurate feedback of force errors is likely to be unavailable to the neural control system (Jones and Hunter, 1982; Jones, 1986; Toffin et al., 2003). The ability to form through practice a representation of compatible force/motion pairs corresponds to representing the environment mechanics in a way that extends the dual concepts of impedance (input motion/output force) and admittance (input force/output motion) beyond the limits of linear analysis.

### Mathematical formulation

Consider the act of manipulating an object. Two sets of independent generalized coordinates describe the configuration space of the arm and of the object. To begin, arm and object have typically a different number of configuration variables. In first approximation, 7 angular coordinates $q\;=\;{\left[q_{1},q_{2},\ldots ,q_{7}\right]}^{T}$ describe the configuration of the human arm. In contrast, six coordinates -$x\;=\;{\left[x_{1},x_{2},\ldots ,x_{6}\right]}^{T}$—or less may be sufficient to uniquely identify the configuration of manipulated objects, such as a hammer or a pencil.

When object and arm are considered separately, two uncoupled systems of ordinary differential equations (ODEs) describe their respective behaviors:

(1) $$\begin{array}{l} \left\{ \begin{array}{c} E\left(\text{s},\dot{\text{s}},\text{F}\right)=0\text{ Object}\\ D\left(\sigma ,\dot{\sigma }\right)=u\left(t\right)\;+\;\tau \text{ Arm} \end{array}\right. \end{array}$$

Here *s* = [*x*, ẋ]^*T*^, and $\sigma =\;{\left[q,\dot{q}\right]}^{T}$ are the state vectors of the object and arm, respectively. The terms F and τ represent externally applied forces (a force/torque vector for the object and joint torque vector for the arm). The arm equation has an additional input term, *u(t)*, representing the neural command.

If arm and object are coupled, the respective configuration spaces are joined by a forward-kinematics map, *x* = *X*(*q*) with Jacobian^3^

(2) $$\begin{array}{l} J\left(q\right)=\frac{\delta X}{\delta q} \end{array}$$

Accordingly, the generalized forces for the object and the arm become related as:

(3) $$\begin{array}{l} \tau =-J^{T}\left(q\right)\cdot F \end{array}$$

Note that in this convention, we are assuming that the object's forces are opposing the internal forces, *u(t)*, generated by the controller.

The environment dynamics, *E*(*s*, ṡ, *F*), define implicitly two functions:

(4) $$\begin{array}{l} \left\{ \begin{array}{c} F=Z\left(s,ṡ\right)\;if\;\frac{\partial E}{\partial F}\neq 0\\ ṡ=Y\left(s,F\right)\;if\;\frac{\partial E}{\partial ṡ}\neq 0 \end{array}\right. \end{array}$$

The two conditions above are in matrix form. Therefore, the inequalities on the right sides represent the requirements that the functional determinants, $\frac{\partial E}{\partial F}$ and $\frac{\partial E}{\partial ṡ}$ be full row rank.

If either condition is not satisfied, then the environment is singular and we have two possible cases:

(5) $$\begin{array}{l} \left\{ \begin{array}{c} ṡ=0\\ or\\ F=0 \end{array}\right. \end{array}$$

The first case describes a rigid constraint (no motion is allowed), the second describes free space (zero interface force).

We make the hypothesis that the neural control system is *perfectly competent* in both cases, i.e., that is capable to generate:

- Desired force trajectories, *F*_*D*_ (*t*) against rigid fixtures
- Desired motion trajectories, *q*_*D*_ (*t*) with *x*_*D*_ (*t*) = *X* (*q*_*D*_(*t*)) in free space.

Perfect competence is insured by the operation of two specialized modules, which we call *singular-controllers*:

(6) $$\begin{array}{l} u_{F}\left(q,F_{D}\left(t\right)\right)=J^{T}\left(q\right)\cdot F_{D}\left(t\right)\text{ Contact Force Controller} \end{array}$$

and

(7) $$\begin{array}{lll} u_{M}\left(\sigma ,\sigma_{D}\left(t\right)\right) & = & D\left(\sigma_{D}\left(t\right),{\dot{\sigma }}_{D}\left(t\right)\right)+\Phi \left(\sigma ,\sigma_{D}\left(t\right)\right)\text{ Motion}\\ \text{Controller} \end{array}$$

with

(7a) $$\begin{array}{l} \sigma =\left(q,\dot{q}\right)\text{ and }\sigma_{D}\left(t\right)=\left(q_{D}\left(t\right),{\dot{q}}_{D}\left(t\right)\right) \end{array}$$

In this expression, we must carefully distinguish between the state vector, σ, and the desired state trajectory σ_*D*_ (*t*). The former is an independent variable, which the controller may estimate based on sensory data. The latter represents an intention, or a “motor program,” and is an explicit function of time. The term Φ (σ, σ_*D*_ (*t*)) is a stabilizing term. Its output depends upon the discrepancy between actual and desired state. This component describes the response of the controller to random perturbations. In fact, earlier work by Won and Hogan (1995) demonstrated that arm movements are dynamically stable. In the following discussion and in the simulations, we will use this simple form for Φ:

(8) $$\begin{array}{l} \Phi \left(\sigma ,\sigma_{D}\left(t\right)\right)=K\cdot \left(q-q_{D}\left(t\right)\right)+B\cdot \left(\dot{q}-{\dot{q}}_{D}\left(t\right)\right) \end{array}$$

In biological control, this stabilizing term arises from the biomechanical properties of the neuromuscular system. The two terms on the right are linearized representations of joint stiffness and damping, respectively. This representation of movement control may be consistent with the equilibrium-point theory (Feldman, 1966, 1986; Bizzi et al., 1984; McIntyre and Bizzi, 1993; Toffin et al., 2003; Wong et al., 2009), where the stabilizing effect is not subject to neural feedback delays. However, here we do not need to make specific assumptions on the form of Φ beyond requiring that it be capable to compensate for random disturbances of the arm's motion. Furthermore, we do not distinguish between the active component of the impedance, associated with neural control, and the passive component, associated with intrinsic viscoelastic properties (which are also modulated by neural activities). We consider motor impedance as a component of the motion control system because of its inherent causality: the damping term limits variations of velocity and the stiffness term limits variations of position. A feedback stabilizing term can also be introduced in the force controller to describe the immediate response to random deviations between actual and measured values of contact force. This could be a subset of proportional, integral, and derivative terms applied to the contact force error. However, unlike for Equation (8), there is no experimental support for such terms in biological control. Therefore, we omit to include a force stabilizer in the model.

### Compatible force/motion pairs

The two singular controllers are competent to generate arbitrary motions in free space and arbitrary contact forces against rigid constraints. A broader goal is to generate arbitrary motions or forces within both singular and non-singular environments. For example, one may perform a movement trajectory within a field of perturbing forces. Or, one may need to apply a constant force against a soft surface rather than a rigid one. In our theory, the control system operates by the concurrent action of the two singular controllers. These attempts, independent of each other, to enforce a pair of trajectories at the interface with their ideal singular environment: a motion trajectory, *x*_*D*_(*t*), in free space and a force trajectory, *F*_*D*_(*t*), against a rigid contact. Together, these trajectories constitute a “target pair,” {*x*_*D*_(*t*), *F*_*D*_(*t*)}, which may not be compatible with the physical properties of the system/environment interface.

In adaptive force control, we start from a desired force *F*_*D*_(*t*) to be applied against a rigid constraint. The singular force controller, (6), is competent to produce this force.

If the rigid constraint is replaced by a non-singular environment the resulting force differs from the desired force, *F* ≠ *F*_*D*_(*t*), and the system moves along a state trajectory *s*(*t*), which satisfies identically the coupled equations:

(9) $$\begin{array}{l} \left\{ \begin{array}{c} ṡ=Y\left(s,F\right)\;\\ D\left(\sigma ,\dot{\sigma }\right)=u_{F}\left(q,F_{D}\left(t\right)\right)-J^{T}\left(q\right)\cdot F \end{array}\right. \end{array}$$

Our hypothesis is that the adaptive controller recovers the desired force trajectory, *F*_*D*_(*t*), by adding to the initial force control policy a singular motion controller, *u*_*M*_(σ, σ_*D*_(*t*)), with $\sigma_{D}\left(t\right)=\left(q_{D}\left(t\right),{\dot{q}}_{D}\left(t\right)\right)$. In this case, σ_*D*_(*t*) is the desired state trajectory of the motion controller, with

(10) $$\begin{array}{l} x_{D}\left(t\right)=X\left(q_{D}\left(t\right)\right) \end{array}$$

where the trajectory *x*_*D*_(*t*) is the (unique) solution of the environment dynamics “driven” by *F*_*D*_(*t*):

(11) $$\begin{array}{l} ṡ=Y\left(s,F\right) \end{array}$$

With this adapted controller *u* = *u*_*M*_(σ, σ_*D*_(*t*))+*u*_*F*_(*q, F*_*D*_(*t*)), the system is reduced to

(12) $$\begin{array}{l} \left\{ \begin{array}{c} ṡ=Y\left(s,F\right)\;\\ D\left(\sigma ,\dot{\sigma }\right)=D\left(\sigma_{D}\left(t\right),{\dot{\sigma }}_{D}\left(t\right)\right)+\Phi \left(\sigma ,\sigma_{D}\left(t\right)\right)-J^{T}\left(q\right)\left(F_{D}\left(t\right)-F\right) \end{array}\right. \end{array}$$

which is identically satisfied by

(13) $$\begin{array}{l} \left\{ \begin{array}{c} F=F_{D}\left(t\right)\\ q=q_{D}\left(t\right) \end{array}\right. \end{array}$$

with ṡ_*D*_ = *Y*(*s*_*D*_(*t*), *F*_*D*_(*t*)).

If the rigid environment is re-established without reverting to the initial force controller, then one expects to observe an after-effect in the force trajectory. This is analogous to the after-effect observed after movement adaptation to a force field, when the force field is unexpectedly removed. No such after effect would be observed if the perturbation were compensated by reducing the contact impedance.

### Motor learning

The derivation above of the motion trajectory that is compatible with the planned force trajectory assumes prior and complete knowledge of the environment dynamics (Equation 11). However, here we assume that there is no such knowledge and describe a simple iterative process through which the discovery of a motion trajectory that is compatible with the planned force trajectory takes place through repeated trials. The proposed approach does not require estimating contact force errors but only the movement following each attempt to produce the desired contact force. We start from the initial condition, in which the environment was rigid. In this case, the compatible pair is

(14) $$\begin{array}{l} \left\{x_{D}\left(=\text{constant}\right),F_{D}\left(t\right)\right\} \end{array}$$

The control system assumes initially that the interface trajectory is a constant—the point of contact—and that the corresponding force is the desired force *F*_*D*_(*t*). Therefore, the combined control system has the initial form

(15) $$\begin{array}{l} {\text{u}}^{\left(0\right)}={\text{u}}_{\text{F}}\left(q,F_{D}\left(t\right)\right)+u_{M}\left(q_{D}=constant\right)\\ ={\text{J}}^{\text{T}}\left(q\right)\cdot {\text{F}}_{\text{D}}\left(t\right)+D\left(\sigma_{D}^{\left(0\right)},\left(0,0\right)\right)+\Phi \left(\left(\text{q},\dot{\text{q}}\right),\sigma_{\text{D}}^{\left(0\right)}\right)\\ ={\text{J}}^{\text{T}}\left(q\right)\cdot {\text{F}}_{\text{D}}\left(t\right)+\Phi \left(\sigma ,\sigma_{D}^{\left(0\right)}\right) \end{array}$$

The last term on the right, Φ(·), is a compensatory element that tends to enforce the constant trajectory $\sigma_{D}^{\left(0\right)}\left(t\right)=\left(q_{D},0\right)$ with *x*_*D*_ = *X*(*q*_*D*_). The superscript in parentheses indicates the iteration trial. The desired trajectory in each trial is the trajectory observed on the previous trial. When the environment changes from stiff to soft, the previous trajectory is the one observed in the stiff environment i.e., is the contact point with near zero velocity.

The iterative adaptation algorithm tracks on trial N the motion observed in trial, N–1:

(16) $$\begin{array}{l} \sigma^{\left(N-1\right)}\left(t\right)=\left(q^{\left(N-1\right)}\left(t\right),{\dot{q}}^{\left(N-1\right)}\left(t\right)\right) \end{array}$$

Then, the desired trajectory at the N-th step is obtained from a convex combination of desired and observed trajectories at the previous step:

(17) $$\begin{array}{l} \sigma_{D}^{\left(N\right)}\left(t\right)=\sigma_{D}^{\left(N-1\right)}\left(t\right)+\lambda \cdot \left(\sigma^{\left(N-1\right)}-\sigma_{D}^{\left(N-1\right)}\left(t\right)\right) \end{array}$$

where 0 < λ ≤ 1 is a parameter that regulates the learning rate. With this, the adaptive controller is

(18) $$\begin{array}{l} \begin{array}{c} u^{\left(N\right)}=u_{F}\left(q,F_{D}\left(t\right)\right)+u_{M}\left(\sigma ,\sigma_{D}^{\left(N\right)}\left(t\right)\right)=\;\\ J^{T}\left(q\right)\cdot F_{D}\left(t\right)+D\left(\sigma_{D}^{\left(N\right)}\left(t\right),{\dot{\sigma }}_{D}^{\left(N\right)}\left(t\right)\right)+\Phi \left(\sigma^{\left(N\right)},\sigma_{D}^{\left(N\right)}\left(t\right)\right) \end{array} \end{array}$$

Thus, the arm dynamics become (see Equation 12):

(19) $$\begin{array}{l} D\left(\sigma^{\left(N\right)},{\dot{\sigma }}^{\left(N\right)}\right)=D\left(\sigma_{D}^{\left(N\right)}\left(t\right),{\dot{\sigma }}_{D}^{\left(N\right)}\left(t\right)\right)\\ +\;\Phi \left(\sigma^{\left(N\right)},\sigma_{D}^{\left(N\right)}\left(t\right)\right)\;+\cdots +J^{T}\left(q\right)\left[F_{D}\left(t\right)-F^{\left(N\right)}\left(t\right)\right] \end{array}$$

Observe that together with Equation (18), this is a recurrent relation for σ and σ_*D*_(*t*).

Therefore, if the trajectories $\sigma_{D}^{N}\left(t\right)$ form a Cauchy sequence, that is if $\sigma^{\left(N\right)}\left(t\right)\to \sigma_{D}^{N}\left(t\right)$ as *N* ↦ ∞, then $F^{\left(N\right)}\left(t\right)\mapsto F_{D}\left(t\right)$ as *N* ↦ ∞. At present, we do not have a general proof that $\sigma_{D}^{N}\left(t\right)$ is a Cauchy sequence. However, in all the tested examples, including those considered here, convergence was reached in a small number of steps.

### Experiment

We investigated how subjects adapt to changes in the environment's mechanics while they are attempting to generate a contact force trajectory. In particular, we considered the task of applying by the hand a time-varying force in the forward direction, *F*_*yD*_, without any lateral component, *F*_*xD*_, reaching a peak of 10 N in T = 1 s:

(20) $$\begin{array}{l} \left\{ \begin{array}{c} F_{xD}\left(t\right)=0\;N\;\\ F_{yD}\left(t\right)=5\left(1-cos\left(\pi t\right)\right)\;N\;with\;0\leq \;t\leq \;T \end{array}\right. \end{array}$$

This task only specified a desired force trajectory and made no requirement about hand motions.

We considered a learning problem, in which, first, a subject's ability to produce this force trajectory against a rigid contact was established. Then, the contact point impedance was suddenly changed from rigid to soft. We considered how this change in contact mechanics affected the produced contact force and if and how the subject learned to re-establish its original desired force trajectory.

We compared the performance of human subjects with a simulations of a computational model derived from the theory.

### Subjects and apparatus

Twelve naive, unimpaired, volunteers (age range: 20–34 yr, gender: 8 male, 4 female) participated in this study after signing an informed consent approved by the Institutional Review Board of Northwestern University (IRB protocol number: STU00026226).

Subjects were comfortably seated on a chair with an adjustable positioning mechanism and held the handle of a robot (HapticMaster, FCS Control Systems), with their right hand. The robot emulated two distinct contact properties: a rigid surface, and a soft surface. Here, by “rigid” we do not intend an ideally rigid contact, with infinite stiffness, but merely a contact with stiffness sufficiently large to prevent significant amounts of motion under the contact forces that are being considered.

A sling attached to the ceiling supported the upper-arm against gravity and arm motions were restricted to the horizontal plane. In the starting position, the elbow and the shoulder joints were flexed approximately at 70° and 50°. The shoulders and wrist were restrained by suitable holders.

### Environments

The stiffness and the damping of the rigid surface were, respectively,

$$\begin{array}{l} K_{e}=\left(\begin{array}{cc} -1000 & 0\\ 0 & -1000 \end{array}\right)N∕m\;B_{e}=\left(\begin{array}{cc} -1000 & 0\\ 0 & -1000 \end{array}\right)Ns∕m \end{array}$$

The stiffness and the damping of the soft surface were

$$\begin{array}{l} K_{e}=\left(\begin{array}{cc} -130 & -52\\ -52 & -70 \end{array}\right)N∕m\;B_{e}=\left(\begin{array}{cc} -28 & -4\\ -4 & -23 \end{array}\right)Ns∕m \end{array}$$

The contact mass was set in both cases to $M\;=\;\left(\begin{array}{cc} 4 & 0\\ 0 & 4 \end{array}\right)\;Kg$. We chose environment parameters compatible with the stability requirements for the robot used in the experiment. The parameters of the rigid environment allowed only minimal motions (<4 mm) of the hand. The eigenvectors of the soft environment were rotated by 60 degrees with respect to the x-y axes because we aimed at inducing a directional effect that could be observed in initial exposures and catch trials.

### Visual display

**Figure 1:**
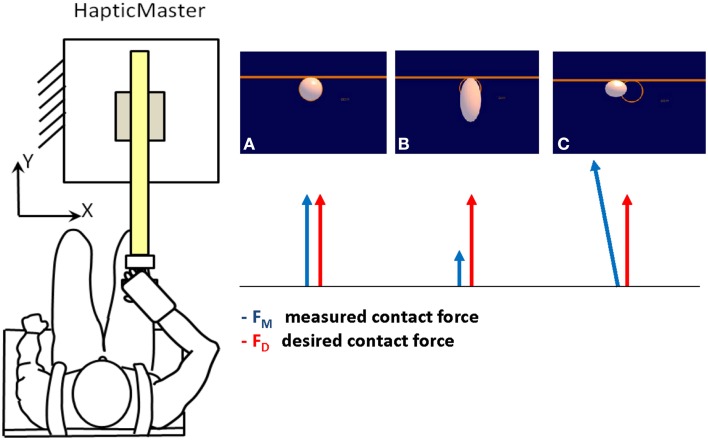
**Experimental set up and visual display**. Subjects held the handle of a high-impedance robot manipulandum (HapticMaster—left panel) and exerted a contact force to preserve the shape and position of a white disk inside an orange ring attached to a bar (right panel). The ring defined the desired position and shape of the disk. Subjects were to exert a contact force in the forward -y- direction to prevent the disk from changing shape. **(A)** The contact force is equal to the desired force; therefore the display is static with a round white disk inside the orange ring. The disk doesn't change shape and doesn't move. **(B)** The measured contact force is smaller than the required one, thus the disk elongates. **(C)** The contact force is bigger than desired, then the disk shrinks. An error in lateral force causes the disk to translate along the bar.

Subjects were provided with visual feedback of the interaction force over a 19” LCD computer monitor. The monitor was placed in front of the subjects, about 1 m away, at eye level. In isometric force experiments, the goal of generating a desired force is typically expressed by displaying the force vector produced by the subject and by presenting a target to be reached. Here, we were concerned with representing the force control task without reducing it graphically to a reaching or a tracking task. To this end, we designed a task in which subjects were to achieve a condition of static equilibrium characterized by the absence of motion. They were asked to exert a contact force to preserve the shape and position of a graphical object on the monitor. The object was a white disk against a blue background. The disk was inside an orange ring attached to a bar (Figure 1, right panel). The ring defined the desired position and shape of the disk. Subjects were to produce a contact force that matched the target pattern (Equation 20).

If successful, the disk did not change shape and did not move along the bar.

The elongation of the disk along the y dimension was a function of the difference between the forward components of the measured force and of the desired contact force. Subjects were to exert a contact force in the forward (y) direction on the manipulandum to prevent the disk from changing shape. Moreover, they were not to exert any force in the orthogonal direction (x): such force component, *F*_*x*_, caused the disk to translate along the bar, in the x direction. The following equation regulated the motion and deformation of the disk:

(21) $$\begin{array}{l} \frac{{\left(x-\alpha F_{x}\right)}^{2}}{R^{2}}+\frac{{\left(y-Rb\right)}^{2}}{{\left(R\left(1-b\right)\right)}^{2}}=1 \end{array}$$

Where $b=\{\begin{array}{c} \beta \left(F_{y}-F_{yD}\right)\;if\;R\left(1-b\right)>\varepsilon \\ 1-\frac{\varepsilon }{R}\;otherwise \end{array}$

The formula describes the implicit equation of an ellipse. *F*_*x*_ and *F*_*y*_ are the measured contact forces in the lateral (*x*) and forward (*y*) direction respectively and *F*_*yD*_ is the desired contact force (Equation 20). *R* (*R* = 25 mm) is the disk radius in the equilibrium condition (*F*_*y*_ = *F*_*yD*__,_), α, β, and ε are constants (7 mm/N, 0.14 N^−1^ and ε = 0.001 respectively). When the contact force is equal to the desired force, the equation describes a circle of radius *R*. When a force *F*_*x*_ is produced in the lateral direction, the disk translates along the x-axis. When the applied and desired contact forces in the forward direction don't match (*F*_*y*_ ≠ *F*_*yD*_) the disk changes shape either elongating (*F*_*y*_ < *F*_*yD*_), or squeezing (*F*_*y*_ > *F*_*yD*_).

A sound prompted the subject to start pushing in order to avoid the changes in the disk's shape. After 1s a different sound indicated that the deformation was finished, the disk disappeared, and the subject could stop applying force. We provided additional information for motivating subjects to exert the required force: the bar and its ring became red if they reached the goal force (10 N) or black, as an alarm, if they overshot the target force by 1/3 of the maximum required (15 N). In the blind trials only the two sounds and the alarm signal were provided.

### Protocol

The entire experimental session lasted about 45 min. The protocol consisted of four phases:

1. **Familiarization:** 40 trials in the rigid environment
2. **Baseline**: 60 trials

53 trials in the rigid environment (48 with visual feedback, and *5* without).

7 catch trials in the soft environment (2 with visual feedback, and 5 without).

3. **Training**: 180 trials

159 trials in the soft environment (144 with visual feedback, and 15 without).

21 catch trials in the rigid environment (6 with visual feedback, and 15 without).

4. **After Effects:** 10 trials in the rigid environment with visual feedback.

In the familiarization phase, subjects learned how to accomplish the task against the rigid environment. The trial duration (*T* in Equation 20) was initially set to 1.5 s for the first 20 trials and then decreased to 1 s.

In the baseline phase, subjects practiced to exert the force trajectories against the rigid environment until reaching the required performance in a stable manner. On few random trials, the manipulandum's admittance was set to be “softer” and visual feedback was suppressed.

During the training phase, the contact was of the soft type with the exception of few random trials (~1/10) where the manipulandum was programmed to be rigid and visual feedback was suppressed. These “catch trials” were introduced to test for after effects during adaptation.

Moreover, we introduced some trials with visual feedback in the same condition where the trials were mostly blind and vice versa, as we wanted to test that the adapted performance did not depend on visual feedback (or lack of).

Finally, the protocol introduced a short target set in the rigid environment (after effects) in order to evaluate how subjects recovered the initial performance, after the exposure to the soft environment.

### Data analysis

Hand position and force trajectories were sampled at 250 Hz. We computed the following performance measures:

- *Maximum Forward Error (MFE):* the maximum force error in the forward direction—i.e., the required force direction.
- *Maximum Lateral Error (MLE):* the maximum force error in the lateral direction—i.e., the force component orthogonal to the required force direction.
- *Maximum Forward Displacement (MFD):* the maximum movement displacement in the forward direction.
- *Maximum Lateral Displacement (MLD):* the maximum movement displacement in the lateral direction

The force error measures reported changes relative to the average force profile at the end of the baseline in the rigid environment.

We investigated how these indicators changed during adaptation to the new environment proprieties. First, we studied if a learning process took place by looking at both forward and lateral errors and investigating if these errors decreased from the onset to the end of training. If the observed reduction of errors were due to reduction of limb impedance at the point of contact, we would observe a small or negligible change of contact force during the catch trials, when the rigid environment was unexpectedly restored. In contrast, if error reduction were a consequence of learned feed-forward control, the unexpected restored rigid environment in the catch trials would generate force errors opposite to the errors observed when the perturbation was first unexpectedly introduced.

### Statistical analysis

To test for learning, we compared the force errors (MFE, MLE) produced in the rigid environment during the baseline with the forces produced against the soft environment in the initial and late phases of training. We performed a One-way repeated measures ANOVA with 3 factors (baseline, initial, and late response). Significant main effects were followed by *post-hoc* analyses (Tukey's test) to determine whether the errors after training were significantly different (decrease) than the errors observed in the initial exposure to the soft environment and were similar to the errors in the baseline (Table 1, upper panel).

Table 1**Maximum Force Errors—MFE, MLE—and Movement Displacement—MFD, MLD—in the forward and lateral direction (mean ± s.d.); clear rows: trials with visual feedback; shaded rows: trials without visual feedback**.

**Table:** 

**FORCE ERROR INDICATORS**							
	**Baseline**	**Initial response**	**Late response**	**Anova**		**Late vs. initial**	**Late vs. baseline**
				*F*_(2, 22)_	***p***	***p***	***p***
MFE [N]	0.39 ± 0.86	−6.26±1.14	0.88 ± 1.83	86.90	<0.0001	0.0001	0.69
MFE [N]	0.48 ± 3.33	−7.33±1.36	−0.23±2.17	43.53	<0.0001	0.0001	0.73
MLE [N]	0.23 ± 0.26	1.54 ± 0.39	−0.12±1.35	12.87	0.0012	0.0012	0.52
MLE [N]	−0.47±0.71	1.5 ± 0.7	−1.51±1.36	31.43	<0.0001	0.0002	0.03

**Table:** 

**CATCH TRIALS**							
	**Early training**	**Late training**	**Anova**			**Late vs. early**	**Late vs. baseline**
			***F***		***p***	***p***	***p***
MFE [N]	–	8.99 ± 4.50	*F*_(1, 11)_ = 46.43^*^		<0.0001		
MFE [N]	13.04 ± 5.98	12.22 ± 6.63	*F*_(2, 22)_ = 19.6		<0.0001	0.93	0.0002
MLE [N]	–	−3.33±0.87	*F*_(1, 11)_ = 182.49^*^		<0.0001		
MLE [N]	−0.5±2.45	−6.29±2.73	*F*_(2, 22)_ = 23.97		<0.0001	0.0001	0.0001

**Table:** 

**DISPLACEMENT INDICATORS**							
	**Early training**	**Late training**	***t*****-test**				
			***p***				
MFD [cm]	4.44 ± 1.03	11.45 ± 1.43	<0.0001				
MFD [cm]	4.12 ± 1.06	11.37 ± 1.84	<0.0001				
MLD [cm]	0.60 ± 0.22	−3.10±0.68	<0.0001				
MLD [cm]	0.64 ± 0.23	−3.56±0.82	<0.0001				

*Force error indicators: One-way repeated measures ANOVA with 3 factors (baseline, initial, and late response). Significant main effects were followed by post-hoc analyses to determine whether the errors after training were significantly different (decrease) with respect to the errors observed in the initial exposure (6th column) to the soft environment and were similar to the errors baseline (7th column). Catch trials: Force errors in the baseline, in the first three and in the last three catch trials with the rigid environment. Significant main effects of the repeated measure ANOVA were followed by post-hoc analyses (5–6th columns)*.

* *only two factors*.

To test for the after effects of adaptation we compared the force errors in the baseline, in the first three and in the last three catch trials with the rigid environment. In this case, significant main effects were followed by *post-hoc* analyses (Tukey's test) to determine whether the errors in the late training were significantly different (increase) with respect to the early training and the baseline (Table 1, medium panel).

We established that the data were approximately normally distributed using the Kolmogorov-Smirnov test and we tested for sphericity using the Mauchly's test. We used the Greenhouse-Geisser procedure to adjust for violation of the sphericity assumption (STATISTICA 7, Statsoft, Tulsa, OK). Significance was accepted at *p* < 0.05.

Understanding the role of vision in force control was beyond the scope of this study. However, to eliminate from our findings all possible bias due to visual feedback, we performed a separate analysis of vision and no vision trials.

The position displacements (MFD, MLD) in the soft environment were a consequence of the exerted force; however, we reported significance (paired two tailed *t*-test) for the changes in these indicators (Table 1, bottom panel).

### Model simulation

We modeled the typical subject's arm as a simplified 2-joint planar arm inertia operated by one- and two-joint muscles. Reasonable values for muscle and limb impedance (stiffness, viscosity, and inertia) during the generation of movement and the application of contact forces were derived from the literature (e.g., Shadmehr and Mussa-Ivaldi, 1994; Gomi and Kawato, 1996; Criscimagna-Hemminger et al., 2003; Damm and McIntyre, 2008) and are equal to the one used by Shadmehr and Mussa-Ivaldi (1994).

The simulated environment was a two-dimensional Mass-Damper-Spring system in a horizontal plane, described by three 2 × 2 matrices (M, Be, and Ke). These matrices were set to have the same values as in the experiment. They were symmetric and positive-definite, to insure passivity. Therefore, the environment was described by 9 (3 × 3) independent parameters. The spring rest-position was always coinciding with the initial position of the arm, to enforce a gradual and smooth application of elastic forces.

The simulation was performed in Matlab, with the ode45 function for integrating the differential equations of the model:

(22) $$\begin{array}{lll} F_{m} & = & K_{e}\left(r-r_{0}\right)+B_{e}ṙ+M_{e}\ddot{r}\\ \text{D}\left(\sigma ,\dot{\sigma }\right) & = & \text{u }-\;{\text{J}}^{\text{T}}\left(\text{q}\right){\text{F}}_{\text{m}} \end{array}$$

where *F*_*m*_ is the contact force measured at the interaction point *r* = [*x y*]^*T*^, *D*(·) the arm endpoint dynamics, *q* the arm configuration, $\sigma =\;\left(q,\dot{q}\right)$ the state, *J* the arm Jacobian and *u* the controller. The initial contact position *r*_*o*_ was set to x = 0, y = 0.3 m from the shoulder joint. For the modular controller, *u* was the combination of a motion control *u*_*M*_ and a force control *u*_*F*_ acting in parallel. This is a particular case of the more general environment/arm coupled dynamics described in the theory above.

## Results

### Simulation results

We evaluated the learning performance of a hybrid system that combines by simple summation the outputs of a motion controller and a force controller. The simulation predicts the motion of the hand and the contact forces in the same conditions tested in the experiment. It derives four different sets of force/motion pairs:

1. Baseline. In this case, the hand is in contact with the rigid surface and the force controller is competent to produce the desired force at the desired contact point.
2. Initial response. This is the response of the two proposed controllers—when the contact mechanics switches from “rigid” to “soft.”
3. Adaptation. The control parameters are gradually changed in the soft contact until the system regains the desired force profile.
4. After-Effects. This is the response of the adapted controller, when the contact mechanics switches back from “soft” to “rigid,” as it was in the baseline.

**Figure 2:**
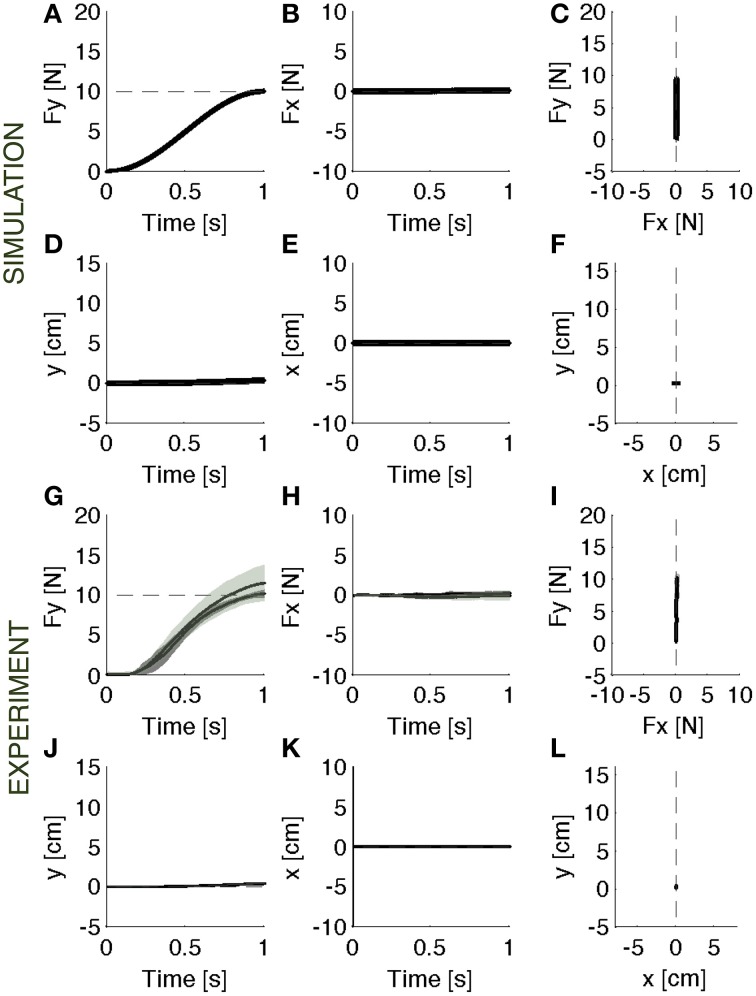
**Baseline**. Contact force profile against a rigid constraint. **(A–F)** Parallel control model results. **(G–L)** Experimental results (mean ± s.d. over all subjects) with (darker intervals) and without visual feedback. The force trajectory follows a smooth temporal profile in the forward direction **(A,G)**, reaching a peak of 10 N, without a significant component in the lateral direction **(B,H)**. As expected, the motion in both forward **(D,J)** and lateral **(E,K)** direction is negligible. The panels on the right **(C,F,I,L)** display the vector plots of the applied forces and endpoint motions. The force vectors grow along the y-axis and the hand position remains confined to the origin.

During baseline, the force controller acts against a rigid constraint and generates the desired force (Figures 2A–F). Since the contact surface is not perfectly rigid, but has a stiffness of 1000 N/m and a damping of 1000 N/m/s, there is a small residual error (<0.03 N) between desired and actual force, and a correspondingly small motion of the contact point (<4 mm).

**Figure 3:**
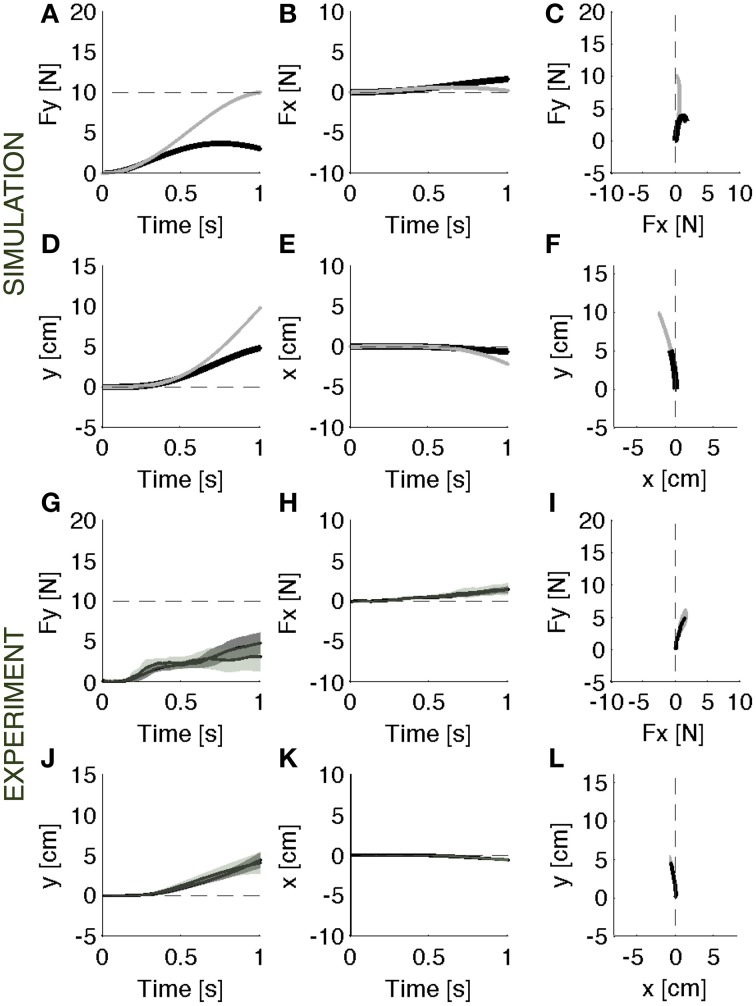
**Initial response**. Contact force profile against the unexpected softer environment. **(A–F)** Black lines: Parallel control model results. Gray lines: the effect of initial exposure to the compliant environment that would result from the force term alone, i.e., without the position control term. It is an ideal force controller that reduces to zero the limb's impedance at the point of contact. **(G–L)** Experimental results (mean ± s.d. over all subjects) with (darker intervals) and without visual feedback. Subjects undershot the target force in the required forward **(G)** direction and an erroneous component on the lateral **(H)** direction as predicted by the model. The results are confirmed by the motion trajectory **(D–F,J–L)**. The panels on the right **(C,F,I,L)** display the vector plots of the applied forces and endpoint motions.

**Figure 4:**
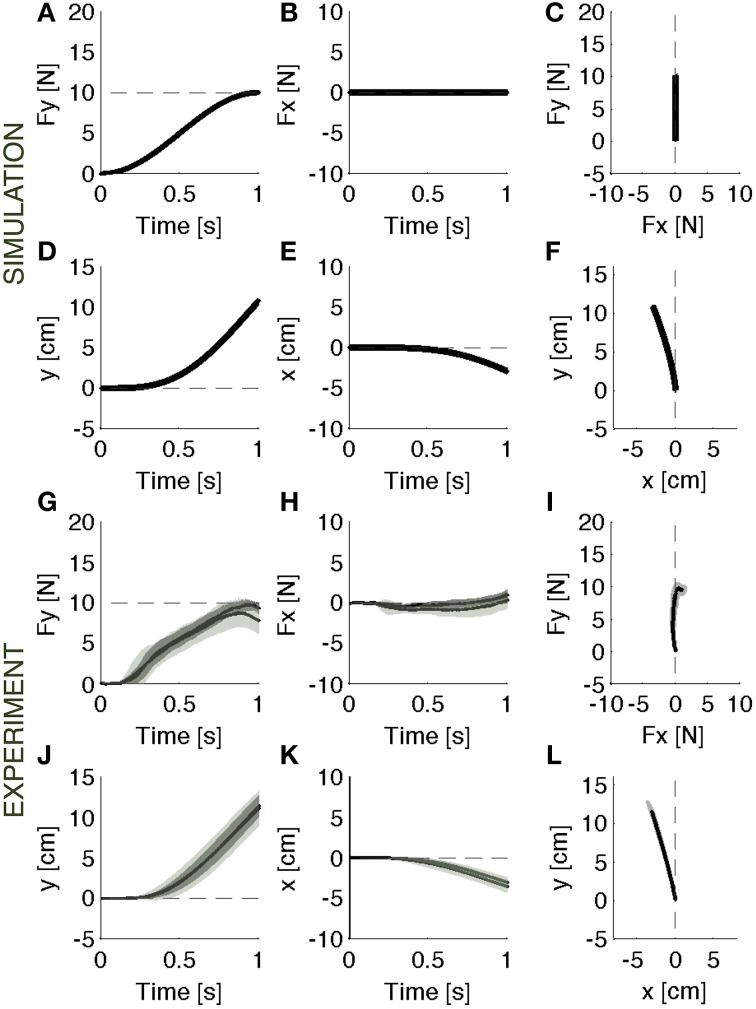
**Adapted response**. Contact force profile against the softer environment. **(A–F)** Parallel control model results. **(G–L)** Experimental results (mean ± s.d. over all subjects) in vision (darker intervals) and no vision condition. The model controller is able to recover the desired force profile **(A–C)** and the corresponding motion trajectory **(D–F)**. The experimental data indicate that subjects, with respect to the initial response phase, were able to generate the required forward force **(G–I)** and the corresponding motion trajectory **(J–L)**. The panels on the right **(C,F,I,L)** display the vector plots of the applied forces and endpoint motions.

When the soft environment is introduced for the first time, the force controller used against the rigid surface does not generate the required force profile as the point of contact moves. In this modular framework, a motion controller acts in parallel with the force controller to maintain a stable contact. As a consequence, when the surface becomes soft, the contact force is reduced by the motion controller, which, in the attempt to resist a displacement of the contact point, effectively adds a force contribution opposite to the desired contact force. This leads to errors both in the forward (Figure 3A) and in the lateral (Figure 3B) directions. In the absence of the motion controller, there would also be a slight reduction of the contact force caused by the motion of the contact point (Figures 3A–C, gray lines). However, this effect would be markedly smaller than the effect associated with the combined systems. In this environment, indeed, the ideal force controller would be almost competent to enforce a desired contact force by canceling the limb's impedance at the point of contact. Therefore, the motion controller with its stabilizing term determines the error in the first interaction with the environment. However, as the environment remains coupled with the body across multiple iterations, the motion controller attempts to compensate the arm/environment dynamics by attracting the arm toward the previously experienced trajectory. This learning procedure gradually corrects the input torque until the desired force is produced within an acceptable error bound (Figures 4A–F).

#### After-effect of adaptation

**Figure 5:**
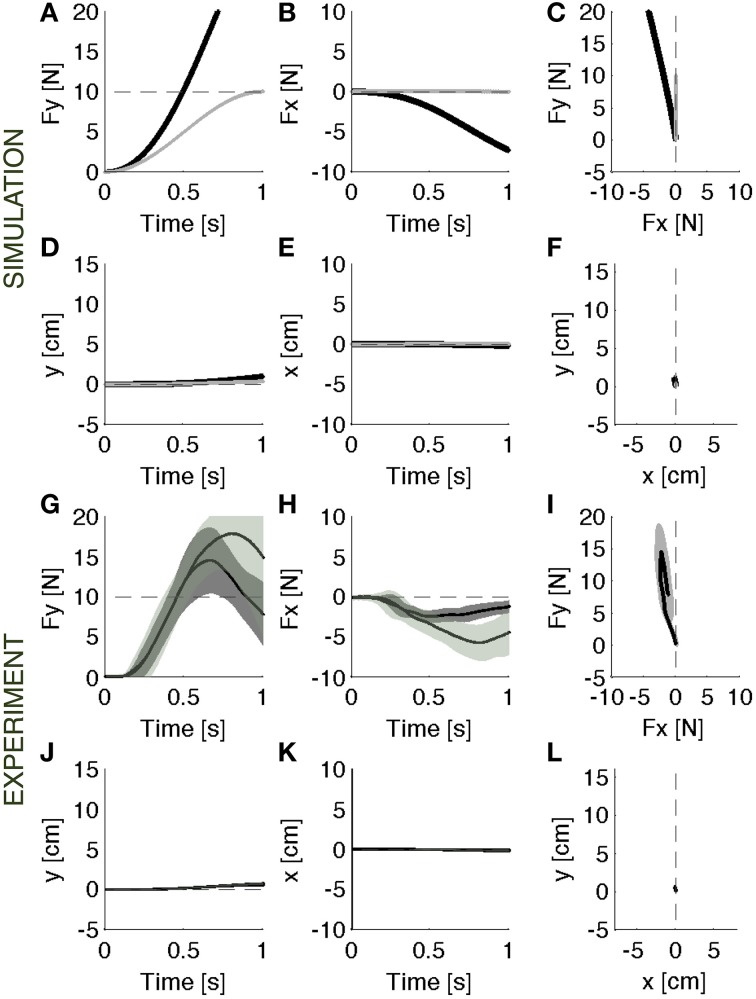
**Catch trials**. Contact force profile against the rigid surface unexpectedly restored. **(A–F)** Black lines: Parallel control model results. Gray lines: the after effects that would result from the same ideal force controller of Figure 3. **(G–L)**: Experimental results (mean ± s.d. over all subjects) in vision (darker intervals) and no vision condition. Subjects operated in the same condition as in baseline, but the achieved performances were different. The force trajectories showed remarkable after effects: subjects overshoot the target force in the desired forward direction **(G)**. In the lateral direction **(H)** they made errors opposite to the error in the initial response to the softer environment. These data are consistent with the predictions of the model **(A–C)**. The motion is negligible in both experiment and model data **(D–F,J–L)**. The panels on the right **(C,F,I,L)** display the vector plots of the applied forces and endpoint motions.

When the rigid environment is suddenly restored, there is an after effect due to the adapted controller that is now unable to induce the previous motion trajectory (Figures 5A–C, black lines). The motion allowed in this environment is smaller than the previously experienced motion (Figures 5J–L). This causes an increase of the output force. Once again, the main contribution to the observed forces comes from the motion controller, which attracts the contact point toward the previous trajectory. The model predicts that after 0.5 s the contact force is twice the desired force of 5N. There is also a lateral error, which is nearly mirror-symmetric to the error experienced in the initial exposure to the soft environment. This is qualitatively similar to the pattern of after effects observed in movement adaptation to force fields (Shadmehr and Mussa-Ivaldi, 1994). The ideal force controller, having negligible contact impedance would not need to adapt to the soft environment, as described above. Therefore, this controller, not having changed, would not predict observing an after-effect (Figures 5A–C, gray lines).

#### Time course of learning

**Figure 6:**
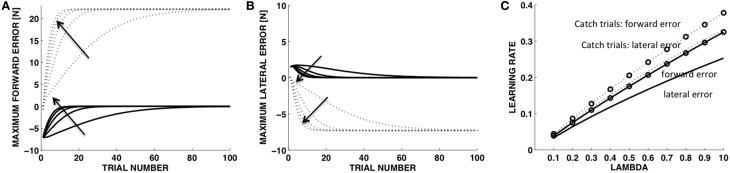
**Model**. Time course of the force indicators with different update rates (λ). Maximum force error in the in the forward direction **(A)** and in the lateral direction **(B)**. **(C)** Learning rate vs. **λ** values. Solid lines: trials in the soft environment. Dotted lines: catch trials in the stiff environment. Arrows indicate increasing values of **λ**.

In the learning model (Equations 14–20) a single parameter, λ (Equation 17), established the extent to which the motion observed in the previous trial contributes to the desired motion in the current trial. Figures 6A,B shows the learning curves corresponding to different values of λ (0.1, 0.3, 0.5, 0.7, 0.9). With λ = 0 there would be no learning at all as the desired motion would not be modified from trial to trial. We fitted to each curve a single exponential to determine the learning rate corresponding to each value of λ (Figure 6C). As expected, the learning rates for all error variables increase monotonically with λ. With the soft contact and in the catch trials, the rates for the forward direction (y) are always greater than the rates in the orthogonal direction (x). However the rates for the catch trials, both in the forward and lateral components, are higher than the rates in the soft environments, with the rate for the lateral component in the catch trials being similar to the rate for the y component in the soft environment. As we show below, these observations are consistent with the experimentally observed learning curves.

### Experimental results

During the baseline, after familiarizing with the task, subjects were required to generate a smooth trajectory of the force in the forward direction, without any lateral force component, reaching a peak of 10 N in 1 second. They learned initially to produce the contact force trajectory against the rigid surface (Figures 2G–I). Then, they practiced reproducing the same force trajectory against the softer surface. We evaluated if subjects were able to recover the desired force profile, by compensating for the motion caused by the force applied to the soft surface.

When the unexpected compliance of the contact point switched to soft, allowing motion to take place, subjects initially undershot the target force and made errors in the lateral—x-direction (Figures 3G–I). With practice, they gradually recovered the desired force profile (Figures 4G–I): with respect to the initial response phase, the force in the required direction increased (y) and the lateral (x) component shrunk. At the end of the training session, some residual force errors were still present, but it was evident that an adaptive process had taken place.

#### After-effect of adaptation

In the analysis of movement adaptation (Flash and Gurevich, 1992; Lackner and Dizio, 1994; Shadmehr and Mussa-Ivaldi, 1994; Gandolfo et al., 1996; Conditt et al., 1997; Thoroughman and Shadmehr, 2000; Scheidt et al., 2001) random “catch trials” were introduced by suddenly replacing the force perturbation with a null-field (i.e., by free space). In contrast, here for the analysis of force-control adaptation we introduced random catch trials by suddenly substituting the soft contact with the rigid contact. In both cases, the catch trials enabled us to establish if the reduction of errors after a period of practice in the softer environment was due to an enhanced force-feedback control mechanism or to an adaptive change in the feedforward command. If the variations of force caused by the change in contact position were reduced by online force-feedback compensation, one would observe a decrease of contact impedance. This would be equivalent, but dual, to the expected increase of impedance if motion perturbations were compensated by a position and/or velocity feedback mechanism. Alternatively, a deterministic movement perturbation to the contact force can be compensated by a change in the feedforward command, resulting in an after-effect when the initial, unperturbed condition is re-established. This latter outcome was evident when the rigid environment was unexpectedly restored at the end of training (Figures 5G–I). Then, subjects overshot the target force in the desired forward (y) direction, and generated an error in the lateral (x) direction. In the catch trials, subjects operated in the same condition as in the baseline, but their performances were clearly different. The force profiles showed strong after-effects of adaptation. In these catch trials, the force errors had opposite sign with respect to the force errors in the initial response to the softer environment. The very existence and size of these after-effects in the experimental data demonstrates that subjects did not decrease their limb impedance, as implied by online force-feedback control. Instead, the after-effects were consistent with the hypothesis of a motion controller acting in parallel with the force controller. The motion trajectories confirmed these observations.

#### Hand motions

When interacting with the rigid constraint, subjects produced a contact force in a quasi-static condition: the hand motion was only about 4 mm (Figures 2J–L). As the experiment switched to the softer environment, the resulting hand motion was about 5 cm, i.e., one order of magnitude bigger than the motion in the rigid environment (Figures 3J–L). During adaptation to the soft contact, the motion trajectories changed length and orientation, gradually converging to a final trajectory—longer than 10 cm—compatible with the desired force (Figures 4J–L).

#### Time course of learning

**Figure 7:**
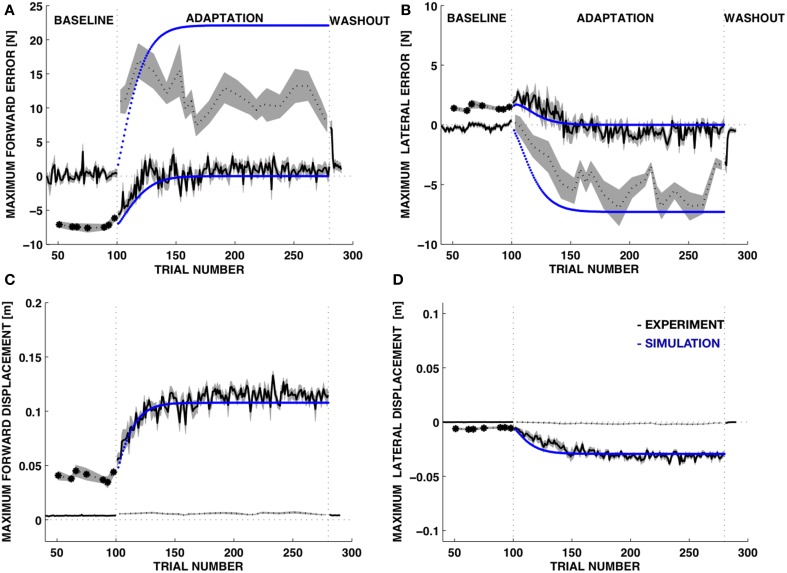
**Time course of the force and motion indicators**. Experimental data vs. model. **(A,B)** Maximum force error (mean±s.e.m.) in the forward (**A**, MFE) and in the lateral directions (**B**, MLE). **(C,D)** Movement displacement (mean±s.e.m. over all subjects) in the forward (MFD) and lateral directions (MLD). BASELINE and WASH OUT: stiff environment; ADAPTATION: soft environment. Black line: experimental data. Dotted line: catch trials, where the environment unexpectedly changes (either from stiff to soft—baseline phase—or from soft to stiff—adaptation phase). Blue line: simulation data.

To quantify these observations, we computed, for each trial and for each subject, the maximum force error and the maximum displacement in the forward (MFE) and lateral (MLE) directions with respect to the subject's reference frame (Figure 7, Table 1). As expected, both force errors (Figure 7, top panels) increased in size when the environment became suddenly softer. While the unknown environment remained coupled with the body, subjects adapted to the new properties of the point of contact. Thus, the force error decreased. The displacement (Figure 7, bottom panels) increased significantly during the adaptation phase and at the end of the adaptation subjects converged toward the motion trajectory compatible with the desired contact force profile.

During catch trials, when the rigid environment was suddenly restored, the forward and lateral force components were significantly different from the baseline. The errors in both directions had opposite sign with respect to those observed in the initial response to the soft environment. While the forward error was immediately large, without relevant changes during the adaptation phase, the lateral error increased significantly with practice. The subjects received an alarm signal when the force exceeded 15 N. This was likely the cause of the rapid error saturation in the forward direction for the catch trials (Figure 7, top-left dotted line).

The learning model of Equations (17–19) was adequate to capture the trends in the force errors and the displacement variables with λ = 0.15, corresponding to 15% update of the desired displacement at each iteration (Figure 7, blue curves). We should stress that this is not intended to be an accurate fitting of the data, but a qualitative account of the learning trends based on a simple learning model with a single free parameter. Experiments on force field adaptation (Donchin et al., 2003; Smith et al., 2006; Scheidt and Stoeckmann, 2007; Judkins and Scheidt, 2014) have revealed a rather large range of learning factors, depending on the environment and the task requirements. However, in our case the learning factor characterizes a process of adaptation that is not quite symmetric to the adaptation of movements to external force fields. While models of the force field adaptation generate changes in movement controllers based on errors in the predicted interaction forces, here we consider changes in the motion control based on errors between commanded and observed movements. In the dual control model the force controller is not updated, but keeps issuing the same feedforward force-command. All the updates are performed by the motion control system that modifies the desired trajectory.

During adaptation, the MFE decreased more rapidly than the MLE and this trend was consistent with a faster increase of the MFE in the catch trials, when the rigid contact was unexpectedly restored. The simulation of the adaptive learning captured this asymmetry. Importantly, the parameter determines different rates of adaptation for the forward and lateral component of the force and predicts a faster increases of the MFE and MLE in catch trials compared to trials in the soft environment (Figure 6C). However, our model cannot reproduce accurately the data in the catch trials because it could not include the effects of the alarm at 15 N.

Taken together, these observations support the hypothesis that adaptation was determined not by decreasing the hand stiffness, but by a motion controller acting in parallel to the force controller. These results were further supported by the separate analysis of the trials without visual feedback, where the adaptive process clearly took place, although there were some residual errors at the end of the training.

Learning occurred for all subjects and was fast: after less than 50 trials both errors were strongly reduced. During the wash out phase subjects recovered rapidly the baseline performance. The after effects decay was faster than the learning time constant, as observed also with adaptation of reaching movements to perturbing force fields (Shadmehr and Mussa-Ivaldi, 1994).

## Discussion

While most studies of motor control deal with the production of movements, here we considered the less explored domain of force control. Force is a classical concept of mechanics, with a rather abstract nature. Unlike motion, force is never directly observed. Instead, force is inferred indirectly from the observation of movement—e.g., the displacement of a strain gage - or of the lack of movement—e.g., the static equilibrium of a balance weight scale. Nevertheless, since Newton the concept of force has proven to be essential for understanding dynamical behaviors. But, is force also an entity represented in the brain? Our results, among others (Lacquaniti and Maioli, 1994; Morris et al., 2007; Chib et al., 2009; Melendez-Calderon et al., 2011), suggest that this may indeed be the case. We have presented experimental evidence and theoretical arguments supporting the hypothesis that to control the interactions of the arm with the environment, the neural system combines the operation of two independent modules: one enforcing desired hand movements in free space and the other controlling the application of desired contact forces against rigid surfaces. The theoretical analysis demonstrates that the concurrent action of these two modules is competent to generate a repertoire of movements and forces in tasks where the hand is in continuous and direct contact with the environment and the CNS corrects for state-dependent deterministic changes of the environment's dynamics.

We investigated the adaptation of force control in an experimental paradigm analogous to the study of movement adaptation within perturbing force fields (Flash and Gurevich, 1992; Lackner and Dizio, 1994; Shadmehr and Mussa-Ivaldi, 1994; Gandolfo et al., 1996; Conditt et al., 1997; Thoroughman and Shadmehr, 2000; Scheidt et al., 2001). We asked subjects to exert a specified force profile against a rigid contact point. As the contact became unexpectedly mobile and compliant, an error appeared in the contact force. After repeated practice, subjects were able to consistently reduce this error. As in the study of movement adaptation to force fields (Shadmehr and Mussa-Ivaldi, 1994), here too the observation of after-effects suggests that adaptive learning was contingent upon the subjects forming a predictive representation of the causal connection between applied forces and resulting motions. This leads to issuing a feedforward command that is maintained when the rigid contact is unexpectedly restored.

The existence of neural representations of contact forces is supported by observations by Kurtzer and coworkers of neural activities in motor areas of non-human primates engaged in maintaining the hand at equilibrium against a load (Kurtzer et al., 2005). They found neurons that express load-related activity either only during the maintenance of arm posture or only during movement.

Venkadesan and Valero-Cuevas (2008) provided further evidence in human subjects for separate neural control of movement and contact force. They demonstrated that during the execution of a tapping task, patterns of muscle synergies switched immediately before a transition from movement in free space to contact with a hard surface. Chib et al. (2009) observed selective disruption of movement control, but not of force control, by transcranial magnetic stimulation (TMS) of posterior parietal regions. This is consistent with earlier studies of posterior parietal cortex, where TMS disrupted oculo-manual interactions (Van Donkelaar et al., 2000). Mugge et al. (2009) studied how our proprioceptive sensory system weights force and position feedback in an environment with known stiffness. They found that position feedback has dominant influence when interacting with soft objects. In contrast, force feedback becomes prevalent with increasing object stiffness. The same group (Mugge et al., 2010) developed a rigorous model of reflex function indicating that position and force feedback are flexibly tuned to position and force tasks.

As neural representations of forces and motions are both expressed by cortical activities, the laws of mechanics establish that arbitrary motions and forces cannot be enforced simultaneously (Spong and Vidyasagar, 1989). Approaches to hybrid control in robotics (Raibert and Craig, 1981; Yoshikawa, 1990) deal with this fact by partitioning the operational space in orthogonal subspaces that allow either motion freedom or force freedom. In this case, two distinct controllers—one for the desired motion and the other for the desired force—are applied independently over orthogonal directions. This approach is applied to tasks such as scraping a rigid surface, where the main computational challenge is to orient appropriately the directions of action for the force and motion controllers. However, in the most common manipulation tasks one interacts with environments that are neither infinitely compliant nor infinitely stiff. When our hand comes in contact with a non-rigid environment, given a trajectory of the contact point there is one and only one trajectory of the contact force, which is compatible (Maluf et al., 2007) with the coupled hand/environment dynamics. Vice-versa, given a trajectory of the contact force there is one and only one compatible motion trajectory. This simple yet fundamental causality principle provides us with the basis for the analysis of adaptive behaviors. Here, we applied this principle to recast the problem of motor learning within a predictable mechanical environment as a search for mutually compatible motion and force trajectories.

Other studies have demonstrated the ability of subjects to learn, memorize, and reproduce patterns of contact forces against a rigid environment (Morris et al., 2007). However, we are not aware of any earlier work in which subjects learn to control and maintain forces against a soft contact point. In this case, the contact point moves so that the motion and force form a unique compatible pair. Melendez-Calderon et al. (2011) performed an experiment, whose results are consistent with the representation of learning as the discovery of compatible forces and motions. In their case, subjects were required to execute hand movements along a simulated “mechanical channel.” This was a typical control framework (Raibert and Craig, 1981; Yoshikawa, 1990), where contact forces were generated perpendicular to the channel walls, which were rigid, and free motions were parallel to the walls. Subjects were presented with a cursor, whose motion was determined in part by the forces detected on the walls of the channel. A dynamic model was used to translate the sensed forces into a movement perturbation that was combined with the hand motion along the channel. Therefore, Melendez's paradigm created a virtual compatible pair of force and motion trajectories and their findings demonstrated that subjects learned through practice to generate such compatible pair. In their case, as in other studies of force control, the force was applied to a rigid constraint and the compatible motion that was displayed to the subject did not correspond to the physical motion of the hand. Instead, it was derived computationally by simulating a dynamic interaction where the same forces would be observed in a non-rigid environment. Thus, their task was not an actual force control task. In contrast, in our study, the compatible pair was physical in that the actual contact impedance established the force/motion correspondence and, unlike with the virtual channel, the direction of the applied forces was not restricted to be orthogonal to the direction of motion.

Adaptation of force control by predictive compensation of the contact mechanics was not the only plausible outcome in our experiments as in force field adaptation. In principle, an alternative response could have been obtained by a force control system reducing the limb's impedance at the point of contact. This would limit the variations of contact force caused by motions of the hand and would not require any computational representation. Instead, this would require the ability to reduce the passive dynamical properties of the arm by effectively lowering the contact impedance through direct feedback of force errors. In our force control task, the presence of after-effects rules out this hypothesis. A controller that minimizes impedance at the point of contact would result in zero or negligible after-effects. When the environment is not predictable, the neural controller can use feedback to modulate motor impedance (Schöner et al., 1992; Franklin et al., 2007; Selen et al., 2009). However, the efficacy of biological feedback is limited by noise and long neural delays (Hogan et al., 1987). Impedance control (Hogan, 1985b; Schöner et al., 1992; Franklin et al., 2003; Damm and McIntyre, 2008) allows for instantaneous responses, but has some well-defined range limitations. On one hand the muscles can only achieve a moderate amount of rigidity and at high metabolic cost (Foley and Meyer, 1993; Hogan et al., 1998; Sih and Stuhmiller, 2003; Franklin et al., 2004). On the other hand the impedance cannot be lowered beyond limits established by the passive mechanics of the musculoskeletal apparatus.

Furthermore, in the biological system, the generation of muscle forces is normally associated with increased stiffness (Hoffer and Andreassen, 1981; Mussa-Ivaldi et al., 1985; Kirsch et al., 1994; Gomi and Osu, 1998; Perreault et al., 2004) and this is likely to further limit the ability of the motor system to operate as an ideal force controller. A possible remedy could be provided by an adaptive force controller acting in conjunction with a motor impedance. But motor impedance is effectively a movement controller that responds with a restoring force to a change in the state of motion. In this case the force controller and the motion controller would act in opposition. The force controller would modify the nominal force based on the experienced force error with respect to the desired force, while the motion controller—the impedance—would attempt to maintain the nominal contact at rest. This approach would eventually lead to recovering the desired contact force and would generate after-effects compatible with those observed in our experiment.

In our model, the path to adaptation is different. The force controller maintains the nominal force at its desired value. It keeps operating as if acting against a rigid environment, while the motion controller updates its nominal position so as to track the motion observed in previous trials. This accomplishes two key results. First, it adjusts the contact force without requiring an accurate estimate of the contact force error that is unlikely available to the biological systems (Jones and Hunter, 1982; Jones, 1986; Toffin et al., 2003). Here, all feedback control is assumed by the motion control system, based on a combination of proprioception and biomechanical impedance. The second, and perhaps most important result from a computational perspective, is the formation of an explicit representation of compatible force/motion pairs given the contact impedance of the environment. In our case the force trajectory is simply the desired trajectory and the compatible motion trajectory is the nominal trajectory of the motion controller. These data, together, constitute the basis for forming a local representation of the environment mechanics.

This approach is similar to the biomimetic controllers proposed by Ganesh et al. (Burdet et al., 2010; Ganesh et al., 2010, 2012) for simultaneous adaptation of force, impedance, and trajectory in interaction tasks. While the implementations of their and our models have significant differences, we share the idea that adapting a reference trajectory can solve these tasks. Their last implementation (Ganesh et al., 2012) suggests moving the reference point of the motion trajectory toward the actual hand position, while simultaneously maintaining the contact force near the specified value attainable with minimum limb impedance.

To investigate motor adaptation, one needs to devise experimental frameworks in which subjects carry out constrained tasks where the goals are given unambiguously, either in terms of desired movement or desired force. This, of course, is quite different from what one encounters in normal circumstances. Manipulation skills generally involve a combination of force and movement goals and we are often uncertain about the mechanical constraints upon which we operate. Consider for example the simple task of turning a crank. In this case, the motion is constrained to a circular trajectory. In a hybrid control scheme one partitions the space in a motion freedom (along the circle) and a force freedom (along the crank). However, the constraint parameters (the center and radius of the crank) may be poorly known or, if one is in the dark, they may be unknown altogether. Then, it is plausible to suggest that, through practice, one learns to remove the contact forces until one learns to perform circular motions by planning to move along the circular path of the crank while maintaining the contact forces at a minimum. However, existing data suggest otherwise. According to observations by Russell and Hogan (1989) subjects learn to maintain a relatively stable pattern of movements and interaction forces along the crank axis, as they keep turning the crank. This is an example of how, given a simple manipulation task, learning may guide toward the discovery of a compatible pair of movement and force trajectories that satisfy the requirements of the task (turning the crank) while producing comfortable and repeatable motor patterns. Srimathveeravalli and Thenkurussi (2005) adopted a similar viewpoint for the representation of handwriting. They suggested that writing styles result from the “haptic profile” of each individual, which is consistent with the generation of specific force/motion trajectories as learned within a particular mechanical environment (e.g., with a particular pen and writing surface) and then exported to other environments.

More broadly, in manipulation tasks, when it comes to deciding whether setting a movement or force goal, one may remain relatively “agnostic” and allow the controller/environment interaction to find the best compromise for satisfying collateral requirements, such as optimality and comfort.

### Considerations on stability

Stability is often related to the maintenance of a controlled variable against random, unpredictable perturbations. In this work, we are addressing an issue of adaptation to predictable, deterministic perturbations, in a way that is analogous but dual to the adaptation of movements to force fields.

We consider two independent controllers, concurrently acting on state and contact force. There are therefore two types of stability to be considered: state stability and force stability. Each form of stability is related to keeping the key variable (force or state) within a bounded neighborhood of a nominal, desired value. Because of the dual character of forces and motions, it is not possible to enforce both forms of stability at the same time. For example, stable position control consists of maintaining position at a particular value or within a bounded region, despite unpredictable force perturbations. State stability in this case is supported by maintaining a sufficient level of impedance, both in its elastic (stiffness) and dissipative (damping) terms. In the case of force control, force stability may be considered as the ability to maintain the contact force at or near a set value, regardless of state uncertainties. Force stability is supported by maintaining low levels of impedance (so that force is insensitive to variations of state). The production of contact force—particularly when pushing against an object—tends to be a threat to state stability and this is particularly true if the contact force is generated at low impedance, as required by standard force control. In our case, with the dual controller, we are sacrificing force stability in exchange for higher state stability because we are assuming that the perturbations are not random but deterministic. By combining in parallel the output of the (low impedance) force controller with the (high impedance) motion controller, one obtains a higher level of state stability at the expenses of force stability in the face of unexpected perturbations. This situation is similar and dual to what one observes in the adaptation of reaching movements to deterministic force fields. In this case, the force is compensated at low impedance. But, because of this, the state stability is reduced—compared to a pure motion controller with high impedance—if the force perturbation becomes random instead of deterministic.

A cautionary note is needed about the concept of motor impedance. In this respect, one must distinguish between the actual and the measured impedance in the following way. As we stated above, a pure force controller seeks stability against random perturbations by decreasing the actual impedance. Instead, the dual motion controller allows stabilizing for deterministic perturbations by tracking the predicted motion of the contact. Consider a hypothetical scenario in which an experimenter applies motion perturbations to the hand of a subject that is exerting a contact force of required amplitude and direction. If the subject's brain can predict the movement that the experimenter will apply, then it will be capable to anticipate this movement with a stiff motion controller. The experimenter will observe an unchanged force and estimate -erroneously—that the subject impedance is close to zero. Instead, the experimenter would observe high impedance when applying random, unpredictable perturbations—which is what we do by suddenly changing the contact from soft to rigid and looking for an after-effect.

### Conflict of interest statement

The authors declare that the research was conducted in the absence of any commercial or financial relationships that could be construed as a potential conflict of interest.

## Footnotes

^1^In this article we focus exclusively on the environment that is external to the body. The skeletal apparatus can be regarded as an environment upon which the muscles act under neural control. In this case, when the arm moves in free space, the humerus, hand, and radioulnar systems are non-singular “objects” controlled by muscle forces. While one must be aware of this ambiguity in the concept of environment, here we remain focused on interactive behaviors and, specifically, on the ability to act on the external environment and to manipulate objects.

^2^We use the term “force trajectory,” in analogy with motion trajectory, to denote a continuous temporal sequence of force vectors. Also, it is important to distinguish net force and applied force. When we apply a force to a rigid surface, by Newton's third law, the net force at contact is zero, because the applied force—the force that can be measured by a strain gage placed between the hand and the surface—is countered by an equal and opposite reaction force.

^3^In general, because of kinematic redundancy, the configuration, q_*D*_, at the point of contact is not unique. However, here we consider only a two-joint planar configuration of the arm, with well-defined inverse kinematics. Also note that here and in the remainder of the article the lower case x indicates the object/arm contact position variable, while the upper case X(·) indicates the forward kinematic function.
